# Gender equity at scientific events

**DOI:** 10.1002/evl3.49

**Published:** 2018-04-21

**Authors:** F. Débarre, N. O. Rode, L. V. Ugelvig

**Affiliations:** ^1^ Centre Interdisciplinaire de Recherche en Biologie (CIRB) Collège de France CNRS UMR 7241–Inserm U1050 Paris France; ^2^ CBGP, INRA, CIRAD, IRD, Montpellier SupAgro Univ. Montpellier Montpellier France; ^3^ Centre for Social Evolution, Department of Biology University of Copenhagen Copenhagen Denmark

**Keywords:** Academic conferences, diversity, evolutionary biology, equal opportunity, gender discrimination, implicit bias, invited speakers, women in STEM

## Abstract

Although the proportion of women in science, and in evolutionary biology in particular, has substantially increased over the last century, women remain underrepresented in academia, especially at senior levels. In addition, their scientific achievements do not always receive the same level of recognition as do men's, which can be reflected in a lower relative representation of women among invited speakers at conferences or specialized courses. Using announcements sent to the EvolDir mailing list between April 2016 and September 2017, and the symposium programs of three large evolutionary biology congresses held in summer 2017, we quantified the representation of women announced as invited speakers in conferences, congress symposia, and specialized courses. We compared the proportion of invited women to a baseline estimated using membership data of the associated scientific societies, and surveyed organizers to investigate their influence and that of potential gender‐ratio guidelines on the proportion of invited women. We find that the average proportion of invited women is comparable (conferences), significantly lower (specialized courses), or significantly higher (congress symposia) than the current baseline (32% women). It is positively correlated to the proportion of women among the organizers, and it is on average higher for events whose organizers considered gender when choosing speakers than for those whose organizers did not. To investigate the impact of Equal Opportunity guidelines, we then collected longitudinal data on the proportion of invited women at two series of congresses, covering the 2001–2017 period. The proportion of invited women is higher when Equal Opportunity guidelines are announced. Encouraging women to sit on organizing committees of scientific events, and the establishment of visible Equal Opportunity guidelines, thus could be ways to ensure higher number of invited female speakers in the future. Our results suggest that change, if desired, requires deliberate actions.

**Impact Summary**

Thirty years ago, a study highlighted the existence of gender inequity among speakers invited to present their research at a large, annual conference in Ecology and Evolution (Gurevitch 1988). Women were less frequently represented among the invited speakers as compared to contributed speakers, and far less likely to be invited to speak if there were no women among the symposium organizers. Over the last decade, a number of initiatives have been put forward in order to increase awareness and reduce implicit biases against female scientists (e.g., Equal Opportunity guidelines, increased transparency in hiring, databases of female scientists, promotion of female role models). We investigated whether women today face fairer chances of being invited to speak at scientific events. We collected the number and gender of invited speakers and organizers from a large number of scientific events within the field of evolutionary biology; 161 conferences, 67 congress symposia and 88 courses held in 2016–2017, and congress symposia held in the period 2001–2017. We used membership data from three large scientific societies as a reference current baseline of women in the field (i.e., constituting the pool from which potential invited speakers would be drawn). Depending on the type of event, the proportion of women among invited speakers is either comparable (conferences), significantly lower (courses), or significantly higher (congress symposia) than the current baseline. A higher proportion of women among the organizers has a positive, significant effect on the proportion of women among the invited speakers across all types of events. Similarly, we find that gender awareness among the organizers and presence of explicit equal‐opportunity guidelines also increase the proportion of invited women. Our results suggest that changes in the proportion of invited women, if desired, require deliberate actions.

Women are still underrepresented in academic science, despite considerable improvements over the last century (Wellenreuther and Otto 2016). While women represented 42% of PhD graduates of all science disciplines across the 28 countries of the European Union (EU) in 2012, they only made up 33% of researchers in these sectors in 2011 (European Union 2016); similar patterns are observed in North America (Handelsman et al. 2005; Hill et al. 2010). These differences can be due to demographic inertia (lower proportion of women in the past (Hargens and Long 2002)) and to differential transition rates between men and women throughout the academic pipeline (Shaw and Stanton 2012), i.e., a higher rate of withdrawal of women compared to men. Decisions to leave academia may be deliberate or not, due to personal choices, or to negative experiences. Those can be caused by unconscious biases whereby both men and women discriminate against women during hiring (Moss‐Racusin et al. 2012; Reuben et al. 2014), when writing recommendation letters (Trix and Psenka 2003; Schmader et al. 2007) or when students rate male versus female instructors or professors (MacNell et al. 2014; Schmidt 2016; Mengel et al. 2017). Differential progression of men and women through the academic pipeline can also result from differential promotion of men and women's achievements, resulting in women receiving fewer awards (Lincoln et al. 2012) and fewer invitations to speak at scientific events (Isbell et al. 2012; Schroeder et al. 2013; Klein et al. 2017).

Caring about the proportion of female scientists is first and foremost a matter of fairness: women should not be favored, but rather given the same opportunities and recognition as men. There is growing evidence that increased diversity not only benefits minority groups, but science as a whole (e.g., Nielsen et al. 2017), in that it bolsters the generation and scrutiny of new ideas and concepts, and helps to keep a field dynamic and productive (Martin et al. 2014). Unfortunately, discussions and debates regarding the underrepresentation of women at higher levels in academia rely on studies focusing on a limited number of conferences, or on subjective impressions. There is thus a demand for sound quantification of the situation, in order to get a clearer and more comprehensive picture of an entire field of science. Moreover, understanding the relative importance of various demographic and psychological factors contributing to the problem can help increase awareness and, in turn, motivate the implementation of initiatives countering these factors.

Inviting a scientist to speak at a scientific event is a recognition of their expertise and leading role in the field. Comparing the proportion of women among announced invited speakers to their proportion in the scientific community may provide clues on how women are perceived as scientists. Organizers of scientific events are likely to be affected by the same psychological factors as those sitting on hiring committees, prize juries, etc. Ensuring a fair representation of both genders at scientific events is also important for the speakers themselves. Invited speakers are given particular exposure, which can have a critical impact on their academic advancement and can bring them to the attention of potential trainees and collaborators, making them potential role models (Lockwood 2006; Martin et al. 2014).

Previous studies surveying the gender of speakers at scientific events in biology showed that the proportion of invited women was lower than baselines in the field or than the proportion of women with contributed talks or posters (Isbell et al. 2012; Schroeder et al. 2013; Kalejta and Palmenberg 2017), despite the existence of female experts (Klein et al. 2017). Several potentially causal factors were identified. First, the proportion of women at the specific career stage from which invited speakers are mostly chosen (i.e., after the PhD) is less than 50%, thereby introducing an inherent inequality. Second, female scientists may decline invitations more often than men, for reasons such as *(i)* some women being invited to participate in conferences disproportionately more, and hence having to decline invitations more often (Schroeder et al. 2013), *(ii)* childcare duties (Schroeder et al. 2013), as well as *(iii)* a lower tendency for self‐promotion and less self‐confidence (Moss‐Racusin and Rudman 2010; Schroeder et al. 2013). Finally, conscious or unconscious biases among organizers can affect the gender composition of invited speakers. Many studies indeed found a positive correlation between the proportion of female invited speakers and female organizers (Gurevitch 1988; Crowe and King 1993; Isbell et al. 2012; Casadevall and Handelsman 2014; Sardelis and Drew 2016; Klein et al. 2017; Kalejta and Palmenberg 2017). Among those studies that tracked the proportion of female invited speakers over several years, some detected increases over time (Crowe and King 1993; Kalejta and Palmenberg 2017), but some others did not (Schroeder et al. 2013; Sardelis and Drew 2016). Although very informative, these previous studies on female representation at scientific events surveyed either a relatively small number of independent conferences (Klein et al. 2017) or symposia at one or a few congresses (Gurevitch 1988; Crowe and King 1993; Isbell et al. 2012; Schroeder et al. 2013; Casadevall and Handelsman 2014; Sardelis and Drew 2016; Kalejta and Palmenberg 2017). Hence, there is a pressing need for an exhaustive and up‐to‐date overview of the representation of female researchers at scientific events of an entire field. We focus on evolutionary biology as a case study, as our own research is anchored within this field.

Here, we quantify the proportion of women announced as invited speakers at evolutionary biology events. We investigate factors potentially affecting this proportion, in particular the role played by organizers, their gender composition and mindset, and the effect of diversity guidelines. Over 18 months, we collected data on invited speakers and organizers from all scientific events advertised on “EvolDir” (Evolution Directory, a listserv compiling, among others, announcements of conferences and courses within evolutionary biology), and from all symposia at three large evolutionary biology congresses that took place in 2017. To elucidate factors affecting the proportion of female invited speakers, we sent a questionnaire to the organizers, asking whether they had taken gender or specified Equal Opportunity (EO) guidelines into account when choosing speakers. In parallel, we obtained information on gender and career stage of members from three important evolutionary biology societies, allowing us to evaluate the current baseline proportion of female researchers likely to be invited to speak at scientific events. Finally, to investigate the temporal effects of diversity guidelines, we compared the proportions of invited women at the symposia of two congresses held over the 2001–2017 period.

## Materials and Methods

### DATA COLLECTION

#### Estimation of the current baseline proportion of women

We contacted scientific societies within the field and asked about the demographics of their members, to estimate the available pool of female researchers that could potentially be invited to speak at scientific events. Three societies agreed to share their membership data: the European Society for Evolutionary Biology (ESEB), the American Society of Naturalists (ASN), and the Society for the Study of Evolution (SSE; data are summarized in Table 1). While the ESEB dataset only separates Student and Non‐Student members, the ASN and SSE datasets could be divided into Student, Postdoc and “Faculty” members, the latter comprising all membership categories that are not Student nor Postdoc (i.e., Regular, Life, Complimentary members, etc.). While some scientists are members of multiple societies, we do not *a priori* expect their genders to be biased. Finally, we also obtained membership archives for ESEB (since 2011, Table S3) and SSE (2008 and 2009, Table S4).

**Table 1:** Proportion of female members of three scientific societies in 2016–2017, listed by career stage

	Student	Postdoc	Faculty	Postdoc+Faculty	All members
ESEB	0.54 (461)	NA	NA	0.38 (983)	0.43 (1444)
SSE	0.52 (848)	0.51 (271)	0.31 (1414)	0.34 (1685)	0.40 (2533)
ASN	0.55 (428)	0.51 (108)	0.24 (688)	0.28 (796)	0.37 (1224)

Sample size is indicated between parentheses. The “Faculty” column comprises all membership categories that are neither Student nor Postdoc (Regular, Life, Complimentary members, etc.). ESEB, European Society for Evolutionary Biology; SSE, Society for the Study of Evolution; ASN, American Society of Naturalists. These different societies have different membership categories. SSE and ASN recently introduced a Postdoc category, while ESEB only distinguishes between Student and Nonstudent members. The 32% estimate is the average of ESEB's ”Postdoc+Faculty,” SSE “Faculty,” and ASN ”Faculty.”

#### Conference and course data (2016–2017)

Each month, from April 2016 to September 2017, we downloaded all emails sent to the EvolDir mailing list (http://life.mcmaster.ca/evoldir.html) under the categories “Conferences” and “WorkshopsCourses.” Announcements were excluded if the event had been announced previously (duplicate), did not have invited speakers, was not about evolutionary biology (e.g., general computing course), or if the event was run by a private company. From this dataset, we also excluded ads corresponding to symposia at the 2017 Evolution ASN and SSE Spotlight Sessions, Society for Molecular Biology and Evolution (SMBE) and ESEB congresses; they were included in a second dataset (“Congress symposia” dataset, cf. section below). In total, we screened 752 ads, of which 249 were included in our study.

For each included event, we counted the number of announced invited speakers, organizers, and women among them, using information from the EvolDir announcement and the event's website. Gender was inferred using the researchers' first names if they were transparent and unambiguous (e.g., John, Jane); otherwise, we searched for pictures on institutional or personal webpages. For simplicity, only binary genders were considered. We focused on the announced invited speakers (i.e., researchers who had accepted the invitation) to evaluate the general stage presence of men and women, i.e., what the audience experiences. For this reason, we did not exclude speakers who were also organizers. Focusing on announced invited speakers rather than people originally contacted by organizers made data collection relatively straightforward, and, importantly, independent of whether organizers had kept a record of all invitations they originally sent. Lastly, we also recorded the country where the event would take place (Fig. S1).

The events were grouped into two categories: Conferences and Courses. The label “Course” was used for events whose main purpose is teaching (88 events), “Conference” for all the other ones (congress, colloquium, annual meeting, etc.; 161 events). For Conferences, the number of invited speakers refers to the sum of all types of invited speakers (e.g., “keynote,” “plenary,” “invited,” etc.); the number of organizers refers to the members of the Scientific Committee, when a distinction between different kinds of organizers exists. For Courses, we also consider the number of invited speakers, or, if this category is absent, the number of instructors. The results for Conferences are presented in the main text, whereas those for Courses are presented in Appendix A.

#### Congress symposium data (2017)

In a separate dataset, we collected information about symposia held at the 2017 Evolution (ASN and SSE Spotlight sessions), SMBE and ESEB congresses (“Congress symposium” data). They were treated separately because the symposia of a given congress are nonindependent events. Data collection took place with the information available on congress websites in February–March 2017. Again, we excluded symposia without invited speakers (e.g., ESEB Open Symposia) and were left with 67 different symposia. For each symposium, we recorded the congress it was part of, the numbers of invited speakers, organizers, and women among each category. As before, we did not exclude speakers who were also organizers (which was the case for 7 (10.4%) symposia).

#### Questionnaires for conference, course and congress symposium data

We contacted the main organizer of each event by email, asking her/him to fill in a short questionnaire accessed via a unique link. On the designated webpage, organizers were first presented with a customized table listing the number and gender composition of invited speakers and organizers collected for their specific event (see Figs. S6 and S7 for screenshots of the survey). They were asked to confirm the numbers (Q0, “Yes”/“No”) or rectify the table. They were then asked whether they were aware of the proportion of women in their final list of invited speakers (Q1, “Yes”/“No”), whether gender was a criterion they had taken into account when choosing whom to invite (Q2, “Yes”/“No”), and whether Equal Opportunity guidelines had been specified regarding the proportion of invited women (Q3, “A given proportion of women was imposed”/“A given proportion of women was suggested”/“No specific guidelines”). A text box was available for comments (Q4; not included in the shared dataset), and lastly the organizer was asked for consent to share their answers (Q5, “Yes”/“No”). If organizers declined to share their answers, which happened in 3 out of 208 events overall, Q1–3 were blinded in the shared dataset. A reminder email was sent to each organizer who had not replied within three weeks, resulting in 65.8% of all emailed organizers responding to our questionnaire.

#### Longitudinal congress symposium data (2001–2017)

For the period 2001–2017, we collected data on the proportion of female invited speakers at the symposia in the ESEB congresses and the SSE symposia of the Evolution congress. The 2001–2011 ESEB data were provided by J. Schroeder and H. Dugdale (Schroeder et al. 2013); all other data were compiled using the available conference programs of ESEB (2013–2017) and Evolution (2001–2017). We then downloaded the entire EvolDir archive, and searched for calls for ESEB and SSE symposia to evaluate whether they stated specific diversity guidelines. We found calls for symposia for all ESEB congresses from 2005 (i.e., for all years available in the EvolDir archive), and calls for SSE symposia for the years 2007–2010, 2012, 2015–2017. In addition, we were provided one SSE call for symposia from 1998 (S.P. Otto, *pers. comm*.).

### STATISTICAL ANALYSES

We did separate analyses of the Conferences (2016–2017), Courses (2016–2017), Congress symposia (2017), and longitudinal congress symposia (2001–2017) datasets, using the lme4 and stats packages (Bates et al. 2015) in R v3.4.3 (R Core Team 2015). We used a binomial error distribution and a logit link function that accounts for differences in the total number of invited speakers among events. All continuous fixed effects (cf. below) were scaled to a mean of zero and standard deviation of one prior to analysis (Schielzeth 2010). We performed backward stepwise model selection using Chi‐squared likelihood ratio tests (Bolker et al. 2009). For each analysis, the 95% confidence intervals based on parametric bootstrap (*n* = 500 replicates) of all tested effects (Bates et al. 2015; Canty and Ripley 2016) are presented in Tables S5–S13. For conciseness, we concentrate on significant effects in the Results section. All datasets and R scripts necessary to reproduce the analyses are available from the Dryad Digital Repository: https://doi.org/10.5061/dryad.nm35n.

For each dataset, we analyzed the proportion of female invited speakers using generalized linear‐mixed models (GLMMs). Potential overdispersion (Harrison 2015) was accounted for by introducing an observation‐level random effect. We tested for an effect of the gender composition of the organizers by including the proportion of female organizers as a continuous covariate. The analysis of the Conference, Course, and Congress symposium datasets included two additional continuous covariates: the total number of organizers (to test if more organizers results in more diverse views and more invited women), and the total number of invited speakers (to test for saturation effects on the proportion of female speakers in large events).

We then analyzed the replies to the questionnaire. First, we tested whether the proportion of female invited speakers affected the organizers' probability of replying to our survey, using a generalized linear model (GLM). Then, we subsetted each of the three datasets to only include events for which a reply was obtained, and tested the effects of the aforementioned covariates as well as the factors corresponding to the answers to survey questions Q1, Q2, and Q3.

We compared the proportion of female invited speakers within each contemporary dataset to our estimate of the current proportion of female researchers in the field using generalized linear models (GLMs). We combined each dataset with the membership data and included a factor with a two‐level fixed effect (collected data vs. society membership data). This test takes into account sampling error in our estimation of the baseline proportion of women by considering the total numbers of society members. For the longitudinal congress symposium datasets, we used the same procedure to compare the proportion of female invited speakers to the proportion of female non‐student members of the corresponding society at the time, whenever membership data were available. Finally, we used the longitudinal congress symposium dataset to investigate a potential increase in the proportion of female invited speakers over time (GLM, with year as a continuous covariate).

## Results

We present the results for the different types of events separately: Conferences and Congress symposia (2016–2017 data and longitudinal data) in the main text, Courses in Appendix A. Results from likelihood ratio tests were in good agreement with 95% bootstrap confidence intervals (see Table S5–S13), except in the estimation how the proportion of women invited effected the probability of organisers replying to our survey (see below).

### CURRENT BASELINE PROPORTION OF WOMEN IN SCIENTIFIC SOCIETIES

Pooling 2016–2017 membership data across the SSE, ASN, and ESEB societies, we obtain an estimated percentage of 32% women potentially invited to speak (this estimate excludes the students from the three societies as well as SSE and ASN Postdocs, see Table 1). This is the current baseline used for comparisons. Note that by including the SSE and ASN Postdoc categories, the estimated percentage of women increases to 34%, while including all categories gives a percentage of 40% women.

### CONFERENCES (2016–2017)

**Figure 1:**
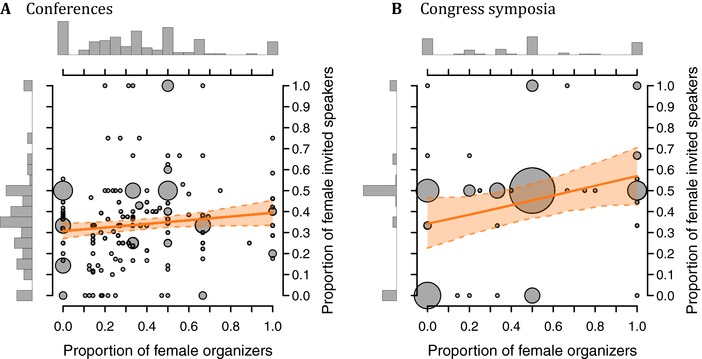
The proportion of female organizers has a positive effect on the proportion of women among invited speakers: (A) in the conference data (*n* = 161 events), (B) in the congress symposia data (*n* = 67 events). The solid and dashed orange lines represent the fitted proportion of invited women and its 95% confidence interval, respectively. The size of the dots is proportional to the number of events.

The distribution of the proportion of female speakers invited at Conferences is shown in Fig. S3A. The estimated average proportion of female speakers across the different Conferences, 34% women, is not significantly different from the estimated current baseline (GLM, *event or society data*, $\chi_{1}^{2}=2.6$, P=0.10). There was a significant positive effect of the proportion of female organizers on the proportion of female invited speakers (Fig. 1 A; GLMM, *proportion of female organizers*; $\chi_{1}^{2}=4$, P=0.044).

**Figure 2:**
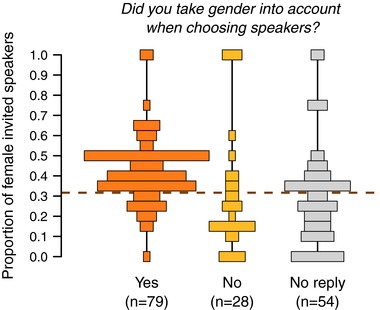
Taking gender into account when choosing invited speakers positively affects the proportion of women among the invited speakers at scientific conferences. The dashed line represents the estimated proportion of women in the field (excluding Student members and ASN‐SSE Postdoc members).

Of the 161 Conference events, we received 109 replies to the questionnaire, of which 107 could be shared and thus included in the analysis. The likelihood that organizers filled in the questionnaire tended to increase with the proportion of females invited to speak at that specific event (Fig. S2A; GLM, *replied to survey*; $\chi_{1}^{2}=6.8$, P=0.0091, but with a 95% bootstrap confidence interval of the estimate overlapping zero, Table S6). This result suggests that the subsample of events for which we received a reply might have been slightly biased. In total, 79 (72%) organizers reported having taken gender into account when choosing the list of speakers (i.e., replied “Yes” to Q2). On average, these organizers invited significantly more women than those who reported not having taken gender into account (Fig. 2; GLMM, *Q2*, $\chi_{2}^{2}=23.9$, $P=6.3\times 10^{-6}$). Moreover, in those 28 events for which the organizer declared not having considered gender, the overall proportion of female invited speakers was significantly lower than the baseline of 32% women estimated from society membership data (GLM, *event vs society data*, $\chi_{1}^{2}=20.7$, $p=5.4\times 10^{-6}$). In contrast, when organizers took gender into account, the overall proportion of female invited speakers was significantly higher than the baseline (GLM, *event vs society data*, $\chi_{1}^{2}=6.5$, P=0.011).

### CONGRESS SYMPOSIA (2017)

The distribution of the proportion of female invited speakers at symposia during the 2017 Evolution (ASN and SSE Spotlight sessions), SMBE, and ESEB congresses are shown together in Fig. S3B. The average proportion of female speakers across the different Symposia (estimated at 45%) is significantly higher than the estimated baseline (GLM, *event or society data*, $\chi_{1}^{2}=11.9$, P=0.00056). As for the conference data, this proportion tended to increase with the proportion of female organizers (Fig. 1 B; GLMM, *proportion of female organizers*; $\chi_{1}^{2}=4.7$, P=0.030).

Of the 67 Congress symposia, we received 46 replies to the questionnaire, 45 of which could be shared and therefore used in the analysis. Again, the contacted organizer tended to be more likely to reply when the proportion of female speakers in the given event was high (Fig. S2B; GLM, *replied to survey*; $\chi_{1}^{2}=4.4$, P=0.036, but with a 95% bootstrap confidence interval of the estimate overlapping zero, Table S12), suggesting that the subsample of events with replies might have been slightly biased. Contrary to the Conferences dataset, we do not find an effect of whether gender was taken into account when choosing whom to invite (GLM, *Q2*; $\chi_{1}^{2}=0.3$, P=0.57), but this might be because only five respondents replied “No.”

**Figure 3:**
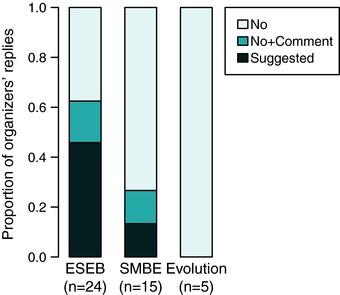
Different perceptions of both the existence and content of equal opportunity guidelines, among symposia organizers at ESEB, SMBE, and Evolution congresses (replies to Q3). Organizer replied “A given proportion of women was suggested” (darkest shade); replied “No specific guidelines” but cited the society's diversity statement in the comment box (intermediate shade); replied “No specific guidelines” and did not comment (lightest shade). One data point was discarded from the ESEB dataset, because the replies were inconsistent. Congress guidelines are available in Appendix B.1.

Unlike the other datasets, the Congress symposia dataset is rather homogeneous, in that all symposia of a given congress adhere to the same Equal Opportunity guidelines (if present; the available Equal Opportunity guidelines are listed in Appendix B.1). Yet, the replies revealed a great disparity in the perception of Equal Opportunity guidelines (Fig. 3). Some organizers declare that no specific guidelines existed (Q3 = “No”), some quoted a diversity statement from either the symposia call or the congress' webpage but replied “No” to the existence of imposed or suggested guidelines (Q3 = “No+Com”), and others indicated that a given proportion of female invited speakers was suggested (Q3 = “Suggested”). These different replies had no effect on the proportion of female invited speakers, suggesting that the organizers' interpretation of the guidelines did not influence whom they chose to invite. Alternatively, the disparity among the replies had a technical cause, namely the wording of the multichoice answers in the questionnaire (see Materials and Methods). Indeed, the diversity statements published with the calls for the Congress symposia (see Appendix B.1) encourage organizers to consider diversity, but do not mention any specific proportion of women.

### LONGITUDINAL CONGRESS SYMPOSIA (2001–2017)

**Figure 4:**
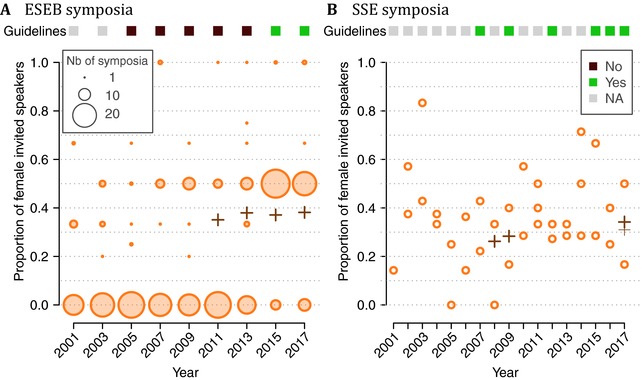
Presence of diversity statements (“Guidelines”) and proportion of female symposia speakers invited at (A) ESEB and (B) SSE congresses. In both panels, the proportion of women among nonstudent members of the corresponding society are shown with crosses (the thinner marker in 2017, panel (B), excludes Postdoc members). For ESEB congresses, the diameter of the circles is proportional to number of symposia. For SSE symposia, each dot corresponds to a single symposium.

To further investigate the role of diversity statement and Equal Opportunity actions, we considered the ESEB and SSE symposia held over the 2001–2017 period (Fig. 4).

#### ESEB

ESEB congresses are usually biennial, and have a large number of symposia (on average 31 symposia per congress in our dataset) with few invited speakers (on average 2.3 per symposium in our dataset). None of the original ESEB calls for symposia available in the EvolDir archive (2005–2017) mention speaker diversity. However, prior to the 2015 congress, an email was sent on EvolDir to encourage gender balance among speakers, and in both 2015 and 2017, diversity statements were published on the congress website (see Appendix B.2). This followed a study pointing out a low proportion of female invited speakers at ESEB congresses (Schroeder et al. 2013), which led to the formation of an Equal Opportunity Committee at ESEB. The increase in the proportion of female invited speakers is likely linked to these events (Fig. 4 A). The proportion of women among invited speakers was significantly lower than the proportion of women among nonstudent ESEB members in 2011 (GLM, *event vs society data*, $\chi_{1}^{2}=12$, P=0.00054), but was not significantly different in subsequent years (GLMs, *event vs society data*, 2013: $\chi_{1}^{2}=2.3$, P=0.13; 2015: $\chi_{1}^{2}=0.4$, P=0.50; 2017: $\chi_{1}^{2}=0.2$, P=0.69).

#### Evolution – SSE

Evolution congresses are annual, with a small number of symposia per society (whereof only SSE were included in this part of our study), but a higher number of invited speakers per symposium than ESEB congresses (on average 7.5 per symposium in our dataset). All calls for SSE symposia found in the EvolDir archive mention speaker diversity, and all but one also explicitly mention gender diversity (see Appendix B.2). No temporal trend in the proportion of female invited speakers was detected (GLM, *year effect*, $\chi_{1}^{2}=0.3$, P=0.58; see Fig. 4 B), and the proportion of female invited speakers was not different from the proportion of women among non‐student SSE members in the years for which we obtained membership data (GLMs, *event vs society data*, 2008: $\chi_{1}^{2}=0.2$, P=0.68; 2009: $\chi_{1}^{2}=0$, P=0.94; 2017: $\chi_{1}^{2}=0.3$, P=0.58).

## Discussion

In this study, we surveyed the proportions of women among invited speakers at scientific events in evolutionary biology, and investigated how the composition and mindset of organizers affects these proportions. The large scale of this study provides an overview of the field of evolutionary biology and helps detect subtle factors that affect the proportion of female invited speakers.

In line with previous studies (Gurevitch 1988; Isbell et al. 2012; Casadevall and Handelsman 2014; Sardelis and Drew 2016; Klein et al. 2017; Kalejta and Palmenberg 2017), we found significant positive correlations between the proportion of women among organizers and the proportion of women among the invited speakers for each type of event we considered (Conference, Congress symposium and Course data; Fig. 1 A, B, and S5). In the absence of precise data regarding organizers' decision processes, we can only speculate about the origin of this effect. It may be caused by unconscious choices (e.g., choosing someone similar to oneself), but also conscious decisions (e.g., women deliberately promoting other women, women being comparatively more willing to accept evidence for gender‐biases in science (Handley et al. 2015) and hence more likely to fight them, or women being more aware of Equal Opportunity guidelines). This could also be because women have more women in their professional networks than men do (Casadevall 2015), or because women are more likely to accept invitations from women than men are (Casadevall and Handelsman 2014). The reciprocal explanations hold true if we interpret our result as male organizers being more likely to invite other men as speakers. While correlation does not imply causation, ensuring that women are represented among organizers may be a simple measure to indirectly promote speaker diversity.

Sardelis and Drew (2016) noted that the number of female invited speakers increased with the total number of invited speakers; while we also observe this effect, we further find a small (albeit non‐significant) negative effect of the number of speakers on the *proportion* of invited women (Fig. S4), as if saturating. Given that women represent 32% of faculty researchers and that they may be more likely to decline invitations than men (Schroeder et al. 2013), it may become harder to come up with a balanced list and to replace women who declined invitations as the number of speakers increases. The use of public lists of female scientists (e.g., “Anne's list” of 350 female neuroscientists (Churchland 2017); “Diversify EEB,” containing over 1200 female ecologists and evolutionary biologists (Duffy and Baucom 2016); “AcademiaNet” with 2445 female academics across all disciplines) could be a way to avoid such a saturation effect.

The Conference dataset reveals that the proportion of female invited speakers is higher when organizers take gender of invited speakers into account. This result shows that the proportion of female invited speakers can be changed through deliberate, conscious measures. Indeed, conference organizers who did not take the gender of invited speakers into account invited on average significantly lower proportions of women than the baseline estimated from society membership data.

Equal Opportunity guidelines may be a remedy to help organizers consider the gender, and more generally, the diversity of the scientists that they invite. However, the results from the Symposia dataset indicate that organizers were not always aware of guidelines edicted by the congresses and ultimately by the scientific societies. To be effective, guidelines should be systematically mentioned in calls for symposia, and be published at a visible location on congress websites. Unfortunately, we cannot exclude that the discrepancy is caused in part by an unclear formulation of Question 3; it asked for the existence of guidelines imposing or suggesting a given proportion of female speakers, while the diversity statements published by the scientific societies merely encouraged organizers to consider (gender) diversity. This technical issue could explain why we found no effect of reported guidelines awareness on the proportion of female speakers in our Symposia dataset. Another reason could be the internalization of guidelines: organizers who are aware of the need to consider diversity may not necessarily pay attention to the existence of specific diversity statements.

The results from the longitudinal symposia dataset corroborates the overall positive effect of Equal Opportunity guidelines specified by scientific societies. All available SSE calls for symposia contain diversity statements, and the proportion of women is not different from the 32% baseline (2016–2017 estimate). ESEB congresses saw a major increase in the proportion of female invited speakers in symposia in 2015 and 2017. Importantly, at least in 2017, this effect was not due to selective acceptance of symposium proposals with higher proportion of females among the suggested invited speakers: the increase was visible even in the submitted symposia (Hannah Dugdale, *pers. comm*.). Schroeder et al. (2013)'s study pointed to the low proportion of female invited speakers at ESEB symposia, and is likely to have played a crucial role in increasing gender awareness in the community, as was the installment of an Equal Opportunity Committee hereafter. This shows that scientific societies – and their members – can play an important role in promoting diversity.

Edicting guidelines raises the question of what the ideal proportion of female invited speakers should be, if quotas were to be installed. A neutral position would require a proportion close to that of women within the given discipline. Determining this proportion is, however, not straightforward, as it varies among sub‐disciplines and over time. For instance, the higher proportion of women among student members of evolutionary biology societies (Table 1) indicates that, even though the academic pipeline is leaky, the proportion of women among senior evolutionary biologists may increase in the future (Shaw and Stanton 2012). Estimating the proportion of women in a field requires the creation, and also the curation, of lists of active researchers. Lists of members of international scientific societies can serve this purpose, because they are topical, regularly updated, and importantly, not restricted to a given country. However, members' gender are not always collected, but could readily be. This way, scientific societies would help provide up‐to‐date censuses of specific disciplines. Alternatively, one may want to promote closer to equal proportion of male and female speakers. The case can easily be made in politics, where individuals are elected and have to represent the general population, of which women constitute half. For scientific events, such a requirement may reflect more personal views on diversity, and may be criticized as being inequitable.

With quotas come the risks of tokenism. Some invited women may feel that they have been invited for their gender and not for their science expertise, while some organizers might claim that diversity is obtained at the expense of excellence. This raises the issue of the subjectivity of defining excellence. Moreover, philosopher Anca Gheaus argues that excellence is never the sole criterion for inviting speakers: their academic reputation also plays a role (but it is an imperfect proxy for merit); so do their connections to the organizers (whose network is necessarily limited and smaller than the entirety of scientists with expertise on the conference's topic), as well as other criteria such as the speaker's sociability. Hence, it is not illegitimate to use gender as an additional criterion (Gheaus 2015). Another attitude, when finding oneself in a token position, consists in actively embracing the opportunity of contributing to normalize the presence of women at scientific conferences, and the responsibility of being a *de facto* role model to younger scientists (Ana Rodrigues, *pers. comm*.).

The representation of women in evolutionary biology and in science in general is inherently linked to the representation of women in the society. Reaching equality between women and men also requires actions at the broader level of the society (Loison et al. 2017). Finally, while our study focused on the representation of women at scientific events, we acknowledge that women are not the only underrepresented category in academia. However, gender equity may be a starting point towards more diversity.

Associate Editor: A. Goswami

## Supporting information


**Appendix A**. Courses dataset.
**Appendix B**. Guidelines.
**Table S1**. Presence of diversity statements in ESEB calls for symposia.
**Table S2**. Presence of diversity statements in the SSE calls for symposia at Evolution.
**Table S3**. Proportion of female non‐student members of ESEB over time; sample sizes are indicated between parentheses.
**Table S4**. Proportion of female non‐student members of SSE over time; sample sizes are indicated between parentheses.
**Table S5**. Estimates (logit scale) and 95% bootstrap confidence intervals of the different effects tested on the proportion of women invited (conference dataset, n = 500 bootstrap replicates).
**Table S6**. Estimates (logit scale) and 95% bootstrap confidence intervals of the different effects tested on reply probability (conference dataset, n = 500 bootstrap replicates).
**Table S7**. Estimates (logit scale) and 95% bootstrap confidence intervals of the different effects tested on the proportion of women invited (subset of conference dataset with replies, n = 500 bootstrap replicates).
**Table S8**. Estimates (logit scale) and 95% bootstrap confidence intervals of the different effects tested on the proportion of women invited (course dataset, n = 500 bootstrap replicates).
**Table S9**. Estimates (logit scale) and 95% bootstrap confidence intervals of the different effects tested on reply probability (course dataset, n = 500 bootstrap replicates).
**Table S10**. Estimates (logit scale) and 95% bootstrap confidence intervals of the different effects tested on the proportion of women invited (subset of course dataset with replies, n = 500 bootstrap replicates).
**Table S11**. Estimates (logit scale) and 95% bootstrap confidence intervals of the different effects tested on the proportion of women invited (symposia dataset, n = 500 bootstrap replicates).
**Table S12**. Estimates (logit scale) and 95% bootstrap confidence intervals of the different effects tested on reply probability (symposia dataset, n = 500 bootstrap replicates).
**Table S13**. Estimates (logit scale) and 95% bootstrap confidence intervals of the different effects tested on the proportion of women invited (subset of symposia dataset with replies, n = 500 bootstrap replicates).
**Figure S1**. Number of ads (Conferences and Courses summed) in the EvolDir dataset, per location country. Dark shade: Conferences, light shade: Courses.
**Figure S2**. Positive effect of the proportion of invited female speakers on the probability to reply.
**Figure S3**. Distribution of the proportion of invited female speakers in the different types of events.
**Figure S4**. (Marginal, non‐significant) Negative effect of the number of invited speakers on the proportion of women among them, in the Conferences dataset (n = 161; GLMM, standardized number of invited speakers; $\chi_{1}^{2}=1.9,p=0.17$).
**Figure S5**. The proportion of female organizers has a positive effect on the proportion of female invited speakers in the Course dataset (n = 88 events.)
**Figure S6**. Screenshot of the survey (April 2016 March 2017).
**Figure S7**. Screenshot of the survey (April 2017 September 2017).
