# Supplementary material for: Gender equity at scientific events
Source: Evol Lett. 2018 Apr 21;2(3):148–58. doi: 10.1002/evl3.49 (PMC6121837; doi:10.1002/evl3.49)
Supplement: Supplementary file 1 — Appendix A. Courses dataset. Appendix B. Guidelines. Table S1. Presence of diversity statements in ESEB calls for symposia. Table S2. Presence of diversity statements in the SSE calls for symposia at Evolution. Table S3. Proportion of female non‐student members of ESEB over time; sample sizes are indicated between parentheses. Table S4. Proportion of female non‐student members of SSE over time; sample sizes are indicated between parentheses. Table S5. Estimates (logit scale) and 95% bootstrap confidence intervals of the different effects tested on the proportion of women invited (conference dataset, n = 500 bootstrap replicates). Table S6. Estimates (logit scale) and 95% bootstrap confidence intervals of the different effects tested on reply probability (conference dataset, n = 500 bootstrap replicates). Table S7. Estimates (logit scale) and 95% bootstrap confidence intervals of the different effects tested on the proportion of women invited (subset of conference dataset with replies, n = 500 bootstrap replicates). Table S8. Estimates (logit scale) and 95% bootstrap confidence intervals of the different effects tested on the proportion of women invited (course dataset, n = 500 bootstrap replicates). Table S9. Estimates (logit scale) and 95% bootstrap confidence intervals of the different effects tested on reply probability (course dataset, n = 500 bootstrap replicates). Table S10. Estimates (logit scale) and 95% bootstrap confidence intervals of the different effects tested on the proportion of women invited (subset of course dataset with replies, n = 500 bootstrap replicates). Table S11. Estimates (logit scale) and 95% bootstrap confidence intervals of the different effects tested on the proportion of women invited (symposia dataset, n = 500 bootstrap replicates). Table S12. Estimates (logit scale) and 95% bootstrap confidence intervals of the different effects tested on reply probability (symposia dataset, n = 500 bootstrap replicates). Table S13. Estimates (logit [file EVL3-2-148-s001.pdf]

Gender equity at scientific events
F. Débarre, N. O. Rode, L. V. Ugelvig

Appendix

A. Courses dataset

The Courses dataset contains 88 events identified as Courses; the distribution of the proportions of invited female speakers for these events is shown on Fig. S3c. The average proportion of female speakers across the different Courses (estimated at 27%) is marginally lower than the estimated baseline (GLM, *event vs. society data*, $\chi^2_1 = 5.4$, $p = 0.020$).

Yet again, we find a significant, positive effect of the proportion of women among the organizers on the proportion of invited female speakers (Fig. S5; GLMM, *proportion of female organizers*; $\chi^2_1 = 8.3$, $p = 0.0039$).

Of the 88 Course organizers we emailed, 53 filled in our questionnaire. In contrast to the Conference and Congress symposia dataset, the propensity of organizers to answer the questionnaire was not affected by the proportion of invited female speakers. Moreover, when we restrict the data to events for which a reply was received, none of the tested factors had an effect on the proportion of invited female speakers.

B. Guidelines

B.1. ESEB, SMBE and Evolution 2017

SMBE

> *The final decision on the selection of symposia, invited and contributed talks will be made jointly by the LOC and International Advisory Board. Criteria will include quality of the proposals, breath of interest to SMBE membership, and representation of the diversity of SMBE membership among speakers.*

Source: `http://www.smbe2017.org/call-for-symposia/`, accessed on 2017-08-03.

ESEB

> *The organizers of ESEB 2017 [. . .] wish to ensure that symposia selected for the meeting in Groningen draw participants (both invited and those selected for inclusion when abstracts are reviewed) as broadly as possible to reflect the full research strengths of the fields represented. We therefore encourage symposium organizers to account for potential implicit biases when selecting among abstract submissions.*

Source: `http://www.eseb2017.nl/programme/symposium-information-/`, accessed on 2017-08-03.

Evolution – SSE

All organizers of the SSE Spotlight Sessions were explicitly asked via email to consider speaker diversity (Aneil Agrawal, *pers. comm.*.)

B.2. ESEB and SSE over time

ESEB symposia

Table S1: *Presence of diversity statements in ESEB calls for symposia. The column "Div.?" indicates whether a diversity statement was present in the call. Excerpts from the diversity statements are provided below. The websites were accessed on 2017-08-28. Unless it is a website address, the call for symposia was found on the EvolDir archive.*

ESEB	Div.?	Source
2001	NA	NA
2003	NA	NA
2005	No	2004-May, `Krakow ESEB Aug15-20 2005`
2007	No	2006-May, `Uppsala EuropeanSocEvolBiol`
2009	No	2008-Apr, `ESEB2009 call for symposia`
2011	No	2010-Jun, `Tuebingen ESEB2011 Aug20-25`
2013	No	2012-Jun, `Lisbon ESEB2013 Aug19-24`
2015	No	2014-Apr, `Lausanne ESEB Aug10-15`
2015	Yes (1)	2014-May, `CallForProposals GenderBias`
2015	Yes (1)	`https://wp.unil.ch/eseb2015/symposium-information/`
2017	No	2016-Jul, `ESEB2017 CallSymposia`
2017	Yes (2)	`http://www.eseb2017.nl/programme/symposium-information-/`

(1) *The organisers of ESEB 2015 [...] wish to ensure that symposia selected for ESEB in Lausanne draw participants (both invited and those selected for inclusion when abstracts are reviewed) as broadly as possible to reflect the full research strengths of the fields represented. We thus encourage prospective symposium organisers to account for potential implicit bias before inviting speakers or submitting their proposals.*

(2) *The organizers of ESEB 2017 [...] wish to ensure that symposia selected for the meeting in Groningen draw participants (both invited and those selected for inclusion when abstracts are reviewed) as broadly as possible to reflect the full research strengths of the fields represented.*

SSE symposia

Table S2: *Presence of diversity statements in the SSE calls for symposia at Evolution. The column "Div.?" indicates whether a diversity statement was present in the call. Excerpts from the diversity statements are provided below. Unless stated otherwise, the call for symposia was found on the EvolDir archive.*

SSE	Div.?	Source
1998	Yes (4)	SP Otto, email `Call for Symposia SSB/SSE 1998 Annual Meeting`
2001	NA	NA
2002	NA	NA
2003	NA	NA
2004	NA	NA
2005	NA	NA
2006	NA	NA
2007	Yes (5)	2006-Feb, `SSEAnnualMeeting CallforSymposia`
2008	NA	NA
2009	Yes (6)	2008-Apr, `UIdahoMoscow SSE2009 Jun13-17 Call for Symposia Proposals`
2010	Yes (6)	2009-Apr, `Portland SSE 2010 CallForSymposia Jun25-29`
2011	NA	NA
2012	Yes (6)	2011-Apr, `Ottawa SSE CallSymposia`
2013	NA	NA
2014	NA	NA
2015	Yes (7)	2014-May, `Brazil EVOLUTION2015 SymposiumProposals`
2016	Yes (7)	2015-June, `SymposiumProposals EvolutionMeeting Austin2016`
2017	Yes (7)	2016-May, `SSE 2017 SymposiumProposals`

(4) *The Council particularly encourages proposals that include speakers from groups traditionally underrepresented in Society symposia, e.g., postdoctoral research associates and new assistant professors, investigators from outside North America, women, and members of racial or ethnic minorities.*

(5) *The Council particularly encourages proposals that include young investigators and others traditionally underrepresented in Society symposia.*

(6) *The Council particularly encourages proposals that include women, younger investigators and others traditionally underrepresented in Society symposia.*

(7) *In evaluating proposals, SSE Council seriously considers the diversity of participants as a major qualification. Symposium organizers are expected to take into account gender, seniority, nationality, and other aspects traditionally underrepresented in Society symposia when preparing proposals.*

Supplementary Figures

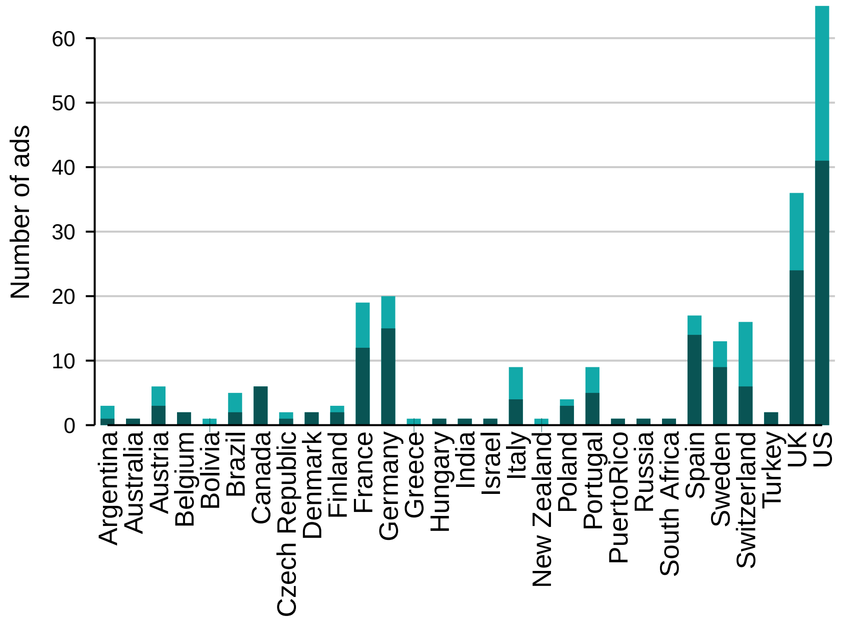

Figure S1: *Number of ads (Conferences and Courses summed) in the EvolDir dataset, per location country. Dark shade: Conferences, light shade: Courses.*

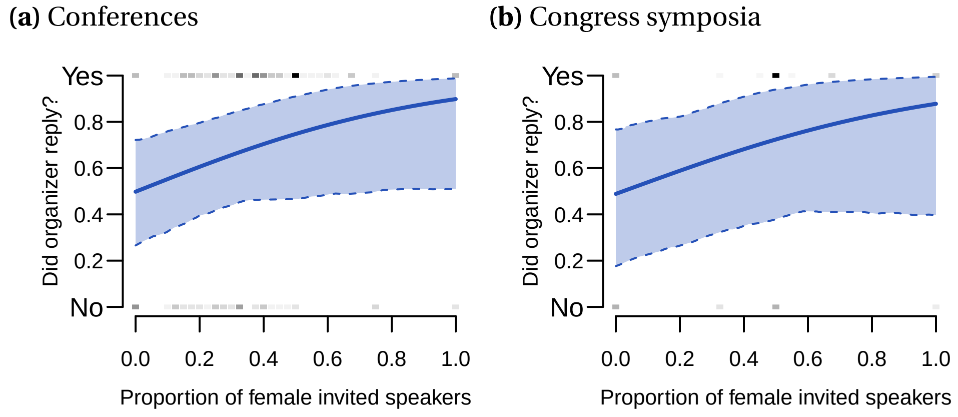

Figure S2: *Positive effect of the proportion of invited female speakers on the probability to reply. The shaded rectangles correspond to one-dimensional histograms of our data, subdivided depending on whether organizers replied or not (i.e., the sum of the two lines of rectangles corresponds to the histograms of Fig. S3).*

(a) Conferences

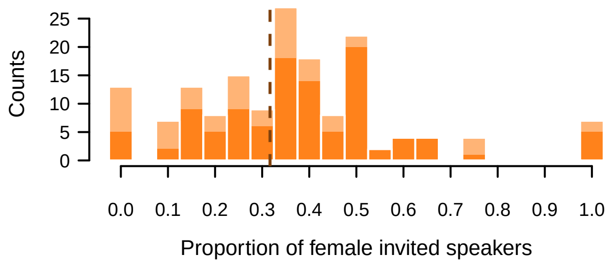

(b) Congress symposia

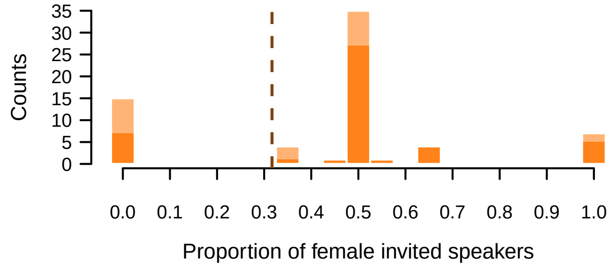

(c) Courses

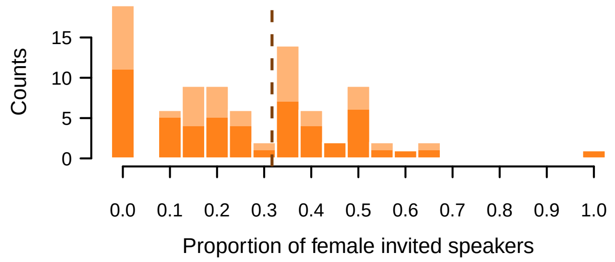

Figure S3: *Distribution of the proportion of invited female speakers in the different types of events. The darker shading corresponds to events for which a reply has been received. The dashed line represents the estimated proportion of women in the field (excluding Students and ASN-SSE Postdocs.)*

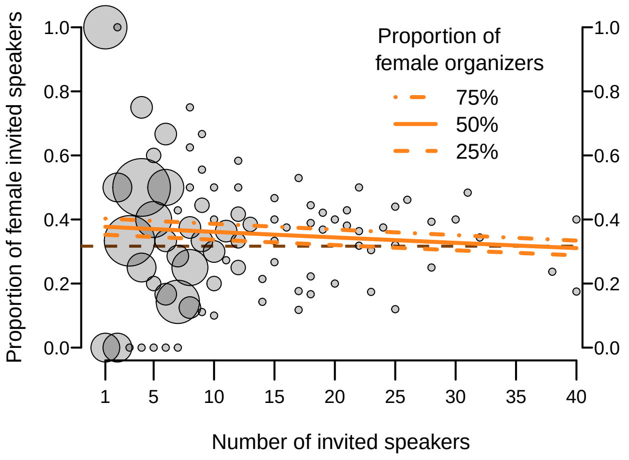

Figure S4: *(Marginal, non-significant) Negative effect of the number of invited speakers on the proportion of women among them, in the Conferences dataset (n= 161; GLMM,* standardized number of invited speakers*;* $\chi^2_1 = 1.9$, $p = 0.17$*). The orange lines indicate the estimated proportion of invited women under different gender compositions of organizers. The brown horizontal line represents the estimated proportion of women in the field (excluding Students and ASN-SSE Postdoc members)*

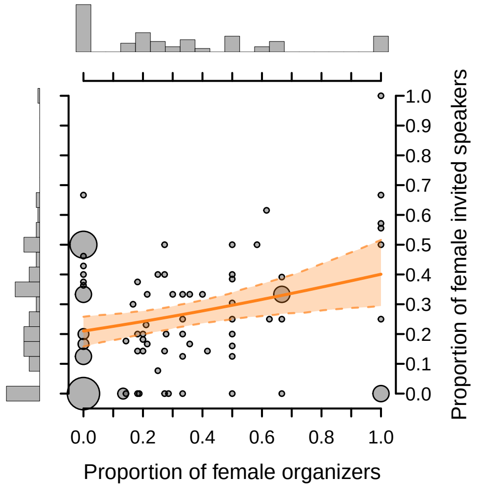

Figure S5: *The proportion of female organizers has a positive effect on the proportion of female invited speakers in the Course dataset (n=88 events.)*

Test_event

Based on the information available in the evoldir email sent on Month 01, and on the conference website, we counted:

#Invited_Total	#Invited_Women	#Organizers_Total	#Organizers_Women
20	9	5	3

Q0 Are these numbers correct?
(Invited = invited and accepted the invitation, i.e., people actually coming to the conference)

- Yes
- No

If there are errors, please change the entries of the table above.

Q1 When you finalized the list of invited speakers, were you aware of what was the proportion of invited women (45 % according to our census) ?

- Yes
- No

Q2 Was the gender of the researchers a criterion that you took into account when you established the list of people to invite?

- Yes
- No

Q3 Were there Equal-Opportunity [EO] guidelines that you had to follow regarding the number or proportions of invited women?

- A given proportion of women was imposed
- A given proportion of women was suggested
- No specific guidelines

Q4 If there were EO guidelines (suggested or imposed), please detail them in the text box (e.g., what proportion of women):
(you can also use this space to send us comments)

Q5 The raw data of the study will be shared on the digital repository Dryad. Do you accept your replies to Questions Q1, Q2, Q3, Q4 to be included in the analysis and hence shared on Dryad?

- Yes
- No

The study will run until July 2017. Please check this box if you wish to be informed about its outcome.

Please click on Submit when you are done. Submit

Figure S6: *Screenshot of the survey (April 2016–March 2017)*

Test_event

Based on the information available in the evoldir email sent on Month 01, and on the event's website, we counted:

#Invited_Total	#Invited_Women	#Organizers_Total	#Organizers_Women
20	9	5	3

NB: "invited" = invited and accepted the invitation, i.e., people actually coming to the event; this can include some organizers.
You can check the list of names that we used to compile the numbers.
We are aware that women may decline invitations more often than men do, but we want to focus on the eventual proportion of female speakers.

Q0 Are these numbers correct?

- Yes
- No

If there are errors, please change the entries of the table above.
Please accept our apologies if we misidentified people. Feel free to send us an updated list of speakers/organizers if they have changed since we collected the data.

Q1 When you finalized the list of invited speakers, were you aware of what was the proportion of invited women?

- Yes
- No

Q2 Was the gender of the researchers a criterion that you took into account when you established the list of people to invite?

- Yes
- No

Q3 Were there Equal-Opportunity [EO] guidelines that you had to follow regarding the number or proportions of invited women?

- A given proportion of women was imposed
- A given proportion of women was suggested
- No specific guidelines

Q4 If there were EO guidelines (suggested or imposed), please detail them in the text box (e.g., what proportion of women): You can also use this space to send us comments, if you wish. In particular, if this is a yearly event and if we already contacted you last year, we are curious to know if our survey affected your choice of speakers this year.

Q5 The raw data of the study will be shared on the digital repository Dryad. Do you accept your replies to Questions Q1, Q2, Q3, Q4 to be included in the analysis and hence shared on Dryad? (if not, we will not be able to use these replies in our analysis)

- Yes
- No

Please check this box if you wish to be informed about the outcome of this study.

Please click on Submit when you are done. Submit

Figure S7: *Screenshot of the survey (April 2017–September 2017)*

Supplementary Tables

ESEB and SSE membership data, previous years

Year	Postdoc+Faculty
2015	0.37 (1013)
2013	0.38 (1103)
2011	0.35 (986)

Table S3: *Proportion of female non-student members of ESEB over time; sample sizes are indicated between parentheses.*

Year	Postdoc+Faculty
2009	0.29 (1684)
2008	0.27 (2001)

Table S4: *Proportion of female non-student members of SSE over time; sample sizes are indicated between parentheses.*

Estimates (logit scale) and 95% bootstrap confidence intervals

Conference dataset

Effect	Type	Estimate	2.5%CI	97.5%CI
Intercept	Fixed	-0.77	-0.97	-0.59
Proportion of female organizers	Fixed	0.38	0.00	0.79
Total number of organizers	Fixed	-0.07	-0.16	0.02
Total number of invited speakers	Fixed	-0.06	-0.18	0.06
Overdispersion (standard deviation)	Random	0.12	0.00	0.30

Table S5: *Estimates (logit scale) and 95% bootstrap confidence intervals of the different effects tested on the proportion of women invited (conference dataset, n = 500 bootstrap replicates)*

Effect	Estimate	2.5%CI	97.5%CI
Intercept	-0.01	-1.02	0.95
Proportion of invited female speakers	2.19	-0.02	4.72

Table S6: *Estimates (logit scale) and 95% bootstrap confidence intervals of the different effects tested on reply probability (conference dataset, n = 500 bootstrap replicates)*

Effect	Type	Estimate	2.5%CI	97.5%CI
Intercept	Fixed	-0.79	-1.81	0.04
Proportion of female organizers	Fixed	0.37	-0.08	0.77
Total number of invited speakers	Fixed	-0.09	-0.20	-0.00
Total number of organizers	Fixed	0.02	-0.17	0.21
Question1=Yes	Fixed	-0.37	-1.07	0.39
Question1=YesNo	Fixed	-0.22	-1.54	1.09
Question2=Yes	Fixed	0.91	0.52	1.34
Question3=No	Fixed	-0.19	-0.89	0.64
Question3=Suggested	Fixed	-0.28	-1.03	0.66
Overdispersion (standard deviation)	Random	0.00	0.00	0.26

Table S7: *Estimates (logit scale) and 95% bootstrap confidence intervals of the different effects tested on the proportion of women invited (subset of conference dataset with replies, n = 500 bootstrap replicates)*

Courses dataset

Effect	Type	Estimate	2.5%CI	97.5%CI
Intercept	Fixed	-1.29	-1.63	-1.02
Proportion of female organizers	Fixed	0.85	0.22	1.62
Total number of invited speakers	Fixed	-0.01	-0.15	0.12
Total number of organizers	Fixed	-0.13	-0.31	0.05
Overdispersion (standard deviation)	Random	0.00	0.00	0.43

Table S8: *Estimates (logit scale) and 95% bootstrap confidence intervals of the different effects tested on the proportion of women invited (course dataset, n = 500 bootstrap replicates)*

Effect	Estimate	2.5%CI	97.5%CI
Intercept	0.20	-0.87	1.16
Proportion of invited female speakers	0.85	-2.37	4.80

Table S9: *Estimates (logit scale) and 95% bootstrap confidence intervals of the different effects tested on reply probability (course dataset, n = 500 bootstrap replicates)*

Effect	Type	Estimate	2.5%CI	97.5%CI
Intercept	Fixed	-0.65	-1.34	-0.02
Proportion of female organizers	Fixed	0.28	-0.63	1.09
Total number of invited speakers	Fixed	-0.03	-0.23	0.17
Total number of organizers	Fixed	0.13	-0.34	0.59
Question1=Yes	Fixed	-0.46	-1.38	0.29
Question2=Yes	Fixed	0.22	-0.31	0.85
Question3=Suggested	Fixed	-0.09	-1.53	0.94
Overdispersion (standard deviation)	Random	0.00	0.00	0.42

Table S10: *Estimates (logit scale) and 95% bootstrap confidence intervals of the different effects tested on the proportion of women invited (subset of course dataset with replies, n = 500 bootstrap replicates)*

Congress Symposia dataset

Effect	Type	Estimate	2.5%CI	97.5%CI
Intercept	Fixed	-0.77	-1.62	-0.13
Proportion of female organizers	Fixed	1.01	0.05	2.20
Conference=Evolution	Fixed	0.80	-0.90	2.37
Conference=SMBE	Fixed	-0.02	-0.87	0.81
Total number of invited speakers	Fixed	-0.15	-0.47	0.14
Total number of organizers	Fixed	0.15	-0.34	0.61
Overdispersion (standard deviation)	Random	0.00	0.00	0.75

Table S11: *Estimates (logit scale) and 95% bootstrap confidence intervals of the different effects tested on the proportion of women invited (symposia dataset, n = 500 bootstrap replicates)*

Effect	Estimate	2.5%CI	97.5%CI
Intercept	-0.05	-1.54	1.19
Proportion of invited female speakers	2.02	-0.56	5.26

Table S12: *Estimates (logit scale) and 95% bootstrap confidence intervals of the different effects tested on reply probability (symposia dataset, n = 500 bootstrap replicates)*

Effect	Type	Estimate	2.5%CI	97.5%CI
Intercept	Fixed	0.76	-1.62	31.10
Proportion of female organizers	Fixed	0.92	-0.26	2.33
Question1=Yes	Fixed	-0.39	-31.62	1.65
Question2=Yes	Fixed	-0.70	-2.66	0.85
Question3=NoButComment	Fixed	-0.87	-2.82	0.49
Question3=Suggested	Fixed	-0.26	-1.25	0.87
Overdispersion (standard deviation)	Random	0.00	0.00	0.82

Table S13: *Estimates (logit scale) and 95% bootstrap confidence intervals of the different effects tested on the proportion of women invited (subset of symposia dataset with replies, n = 500 bootstrap replicates)*
